# Retraction Note: Routine point-of-care ultrasound (POCUS) assessment of gastric antral content in traumatic emergency surgical patients for prevention of aspiration pneumonitis: an observational clinical trial

**DOI:** 10.1186/s12871-022-01759-6

**Published:** 2022-07-21

**Authors:** Mohamed S. Shorbagy, Amr A. Kasem, Ahmed A. Gamal Eldin, Ramy Mahrose

**Affiliations:** 1 Anesthesia and Intensive Care, Faculty of Medicine, Ain Shams University, Cairo, Egypt; 2 Diagnostic and Interventional Radiology Atomic Energy Authority, Faculty of Medicine, Ain Shams University, Cairo, Egypt

**Retraction Note: BMC Anesthesiol 21, 140 (2021)**

**https://doi.org/10.1186/s12871-021-01357-y**

The Editor has retracted this article. After publication the Editor was notified that figures 1-4 [1] and 5-6 [2] had been previously published in other publications and that the authors had not attributed these figures to their sources and had not obtained permissions to reproduce them.

Mohamed S. Shorbagy, Amr A. Kasem and Ahmed A. Gamal Eldin have not responded to any correspondence from the editor/publisher about this retraction. Ramy Mahrose has informed the journal that they have been a victim of identity theft and so they were unaware of this study and its publication. Ramy Mahrose agrees to this retraction.
